# How Thick Aqueous Alkali Should be Better for Aluminum‐Air Batteries at Sub‐Zero Temperatures: A Critical Anti‐Freezing Concentration

**DOI:** 10.1002/advs.202402005

**Published:** 2024-05-30

**Authors:** Hongyu Cui, Ming Gao, Guoqin Cao, Fanfan Liu, Junhua Hu, Jinjin Ban

**Affiliations:** ^1^ School of Materials Science and Engineering State Center for International Cooperation on Designer Low‐Carbon & Environmental Materials (CDLCEM), Zhengzhou University Zhengzhou Henan 450001 P. R. China; ^2^ School of Computational Science and Electronics Hunan Institute of Engineering Xiangtan 411104 P. R. China; ^3^ Key Laboratory of Advanced Energy Materials Chemistry (Ministry of Education) Nankai University Institution Tianjin 300071 P. R. China

**Keywords:** aluminum‐air batterie, critical anti‐freezing concentration, CsOH electrolyte, non hydrogen‐bonds

## Abstract

The application of portable aluminum‐air batteries (AABs) in extreme environments is an inevitable demand for future development. Aqueous electrolyte freezing is a major challenge for low‐temperature operations. Conventionally, enlightened by the organic system in metal ion batteries, blindly increasing the concentration is regarded as an efficient technique to reduce the freezing point (FP). However, the underlying contradiction between the adjusting mechanism of the FP and OH^−^ transportation is ignored. Herein, the aqueous alkali solution of CsOH is researched as a prototype to disclose the intrinsic conductive behavior and related solvent structure evolution. Different from these inorganic electrolyte systems, the concept of a critical anti‐freezing concentration (CFC) is proposed based on a specific temperature. The relationship between hydrogen bond reconstruction and de‐solvation behavior is analyzed. A high conductivity is obtained at −30 °C, which is also a recorded value in an intrinsic aqueous AAB. The homogenous dissolution of the Al anode is also observed. As a general rule, the CFC concept is also applied in both the KOH and NaOH systems.

## Graphical abstract

The intrinsic conductive behavior and solvent structure evolution of CsOH are studied. With the decrease of the CsOH, the HB reconstruction and the Cs^+^ desolvation are occurred. At the CFC, Cs^+^ has a sparse solvated structure that is attributed to the decreased Cs^+^‐OH^−^ and weak non‐HB. The OH^−^ in the first shell layer of Cs^+^ can escape easily and transfer faster, which can be sufficiently diffused to generate homogeneous corrosion.

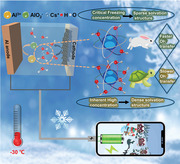

## 1 Introduction

Aluminum‐air batteries (AABs) are a promising electrical energy system due to their high theoretical energy density (8100 Wh kg^−1^ versus zinc‐air batteries 6800 Wh kg^−1^), high safety, portability, and abundant resource (8.1 wt.% in Earth's crust).^[^ ^1^, ^2^, ^3^, ^4^ ^]^ AABs typically use a strong alkaline solution as the electrolyte,^[^ ^5^ ^]^ which exhibits a low freezing point (FP) owing to its colligative qualities.^[^ ^6^ ^]^ The expanded applications in extreme environments, such as the plateau, polar and deep seas, etc., have attracted wide attention.^[^ ^7^, ^8^ ^]^ Despite the benefits of alkali solution‐based AABs at low temperatures, little study has been done on their low‐temperature performance. Electrolyte freezing and lower conductivity are considered typical limiting factors in aqueous electrolyte.^[^ ^9^, ^10^ ^]^

Among previous studies of low‐temperature metal‐air batteries,^[^ ^11^ ^]^ electrolyte modification strategies are deemed as a facile and powerful method for determining the electrochemical reaction inside the AABs, including increased concentration and supplemental additives. Recently, Zhi et al. optimized the concentration of traditional KOH for wide working temperatures.^[^ ^12^ ^]^ As a conventional strategy, the concentrated alkali solution is more effective at resisting icing. It is expected that more solute atoms can disrupt the continuity of hydrogen bond (H‐bonds) between water molecules. KOH solutions with higher concentrations could sustain a liquid state at extremely low temperatures. An alkaline electrolyte with a concentration of 6–8 m, and even higher is always applied irrationally. However, further increasing concentration can't promote conductivity accordingly. To overcome this bottleneck, electrolyte additives were used. Dimethyl sulfoxide could disrupt H‐bonds between zinc‐air battery water molecules, improving freezing tolerance and electrochemical performance.^[^ ^13^ ^]^ However, adding organic additives will increase viscosity and sacrifice conductivity, which will also obstruct the clarification of the intrinsic solution structure.^[^ ^14^ ^]^ Hence, further investigation is needed to disclose how solute atoms interact with OH^−^ transportation.

Herein, we selected CsOH as a prototype electrolyte to investigate the dialectical relationship between electrolyte concentration and ion transmission in the solution. Compared to KOH and NaOH, CsOH has a larger solvated radius, which can improve ion mobility.^[^ ^15^, ^16^ ^]^ Interestingly, the H‐bonds strength and the alkali metal solvation structure are mutated at the critical anti‐freezing concentrations (CFC). AABs show excellent discharge performance based on CFC‐determined electrolyte concentration, which is quite different from the widely accepted high concentration electrolyte of aqueous electrolytes. Regarding the above discoveries, this work studies and discloses the intrinsic aqueous structure evolution with the alkali concentration at sub‐zero temperatures as follows.

## 2 Results and Discussion

**Figure 1:**
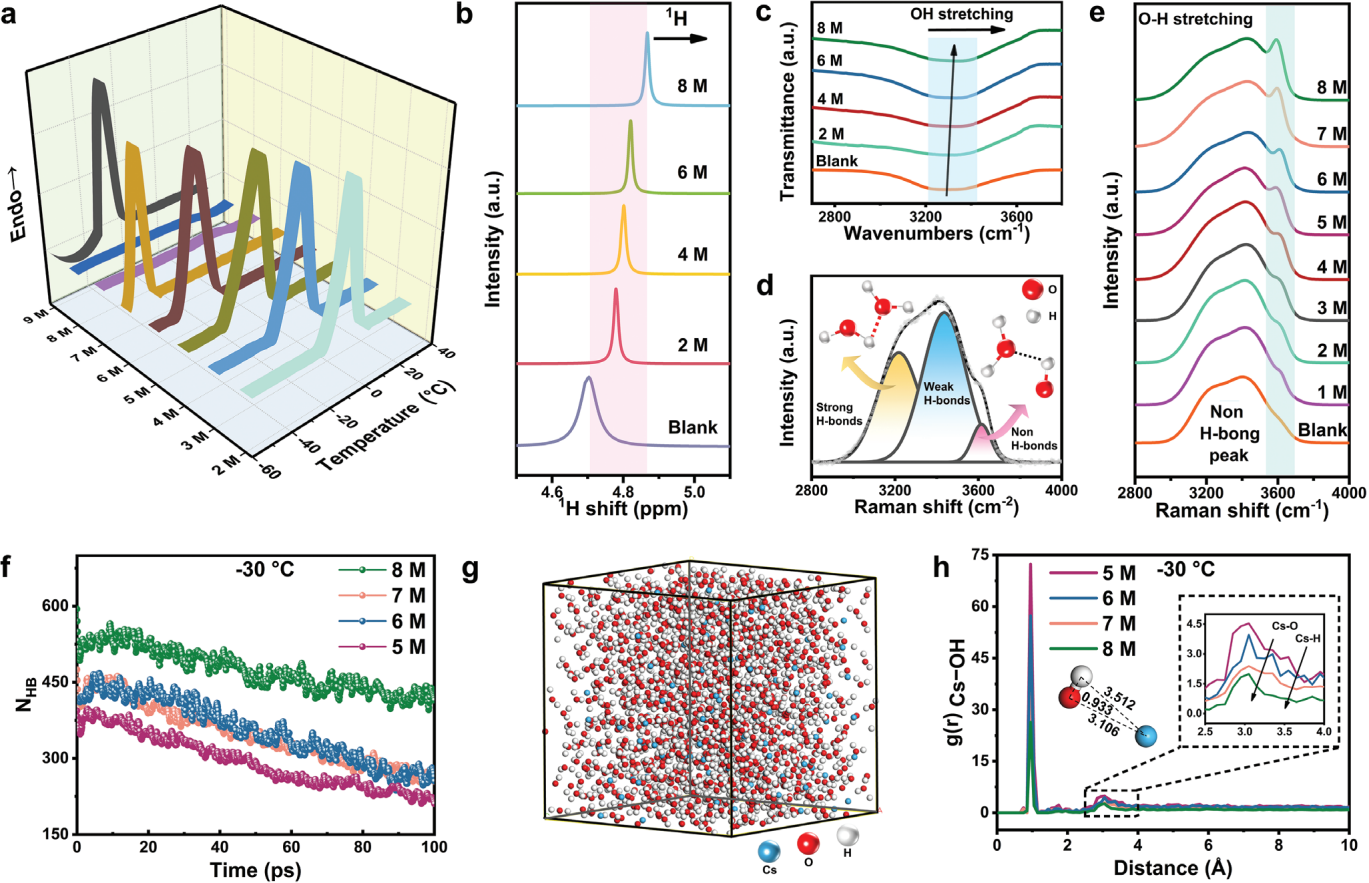
a) DSC for CsOH electrolytes with different concentrations. b) 1H NMR, c) FTIR, and d) The fitted peaks of the O─H stretching vibration. e) Raman spectra of CsOH solutions with different concentrations. f) The average non‐H‐bonds number from MD simulations at −30 °C. g) The MD simulation snapshot box of 5 m CsOH electrolyte. Colors for different elements: H white, O red, Cs blue. h) The Cs‐OH radical distribution function of CsOH electrolytes with different concentrations at −30 °C.

First, the FP of CsOH electrolytes with different concentrations were accurately identified through frozen experiments by differential scanning calorimetry (DSC). As shown in **Figure** **1**a, the solid‐liquid transition temperature of the electrolyte gradually decreases with increased concentration. Notably, the FP of 2–6 m CsOH is −10, −17, −23, −37, and −52 °C (Figure S1a, Supporting Information), respectively. According to the relationship between concentration and FP during battery discharge, the FP may be affected by the generation of reaction products and hydrogen bubbles during AAB discharge. Relative to the black line in Figure S1a (Supporting Information), the concentration has a positive shift of 0.5–1 m. This red line is defined as CFC. The CFCs at −10, −20, and −30 °C are defined as 3, 4, 5 m CsOH. This discovery is visually illustrated in Figure S1b–d (Supporting Information).

For the low‐temperature battery system, performance decline is mainly caused by low ionic conductivity.^[^ ^17^ ^]^ The kinetic theory of molecules illustrates that the shortened distance and enhanced interaction between the centers of the dipole (ion) are the intrinsic causes of this phenomenon.^[^ ^18^ ^]^ The solvation configuration was characterized based on the chemical shift in ^1^H NMR spectra. As shown in Figure 1b, the hydrogen nuclei in H_2_O (≈4.7 ppm) shift to the lower field as CsOH concentration increases, which implies that the electron density of H gradually decreases. The introduction of CsOH solute molecules disrupts the proton shielding from the water molecules, leading to the breakage of the H‐bonds between water molecules.^[^ ^19^, ^20^, ^21^, ^22^ ^]^

In pure CsOH solution systems, where the freely moving molecules and ions are made up only of Cs^+^, OH^−^, and water, the solvation process mainly depends on the interaction of O···H. In FTIR spectra (Figure 1c), the peak at 3200–3500 cm^−1^ is attributed to O─H stretching, in which the redshift suggests the O‐H becomes more asymmetric with the increasing CsOH concentration, mainly caused by the weakened H‐bonds. Such bond changes would promote the formation of the solvated macromolecules (Cs^+^─O─H···H─O─H).^[^ ^23^, ^24^ ^]^ Additionally, the Raman spectra can further display the evolution of the H‐bonds. O─H stretching vibration at around 3000–3700 cm^−1^ can be deconvoluted into three peaks at 3230, 3450, and 3620 cm^−1^ (Figure 1d), assigned to strong, weak, and non‐H‐bonds, corresponding to the symmetric, asymmetric and free O─H stretching vibrations.^[^ ^25^, ^26^, ^27^, ^28^, ^29^, ^30^ ^]^ Figure 1e shows an opposite trend between strong H‐bonds (H─O─H···H─O─H) and non H‐bonds (H─O─H···O─H) as the concentration changes. The content of non‐H‐bonds ratio increases from 4.81% to 10.44% when the concentration of CsOH rises from 0 to 8 m (Figure S2a–i and Table S1, Supporting Information). Contrarily, the strong‐H‐bonds ratio drops from 44.92% to 25.34%. Moreover, the variation pattern of non‐H‐bonds at low temperatures was analyzed by classical molecular dynamics (Figure 1f; Figure S3a,b, Supporting Information). The selected non‐H‐bonds length cutoff was determined to be 2.7 Å,^[^ ^31^ ^]^ where the non‐H‐bonds fraction drops from 8 m (32.27%) to 5 m (20.52%) at −30 °C, consistent with the experimental trend.

Furthermore, the solvation structure of the CsOH electrolyte depends on H‐bonds and Cs^+^─OH^−^ coordination. That is, the de‐solvation process is not only related to the solvent water state but also affected by the adsorption of the solute. The snapshot of CsOH electrolyte solvation structure simulation shows the ionic solvation cluster of Cs^+^, OH^−^, and water molecules (Figure 1g; Figure S4a–f, Supporting Information). Monitored by the radial distribution functions g(r) under low temperatures (Figure 1h; Figure S5a,b, Supporting Information). Hydroxylation around Cs^+^ shows that the introduction of CsOH can form the interatomic bond of Cs─H and Cs─O. Typically, the distances of Cs─O and Cs─H are 3.106 and 3.512 Å, respectively.^[^ ^32^ ^]^ The g(r) value is reduced when the electrolyte is diluted. At −30 °C, the CF is 5 m. Compared with 8 m concentration, it is clear that a sparse solvation structure with weaker Cs^+^─OH^−^ interactions will be obtained.^[^ ^33^ ^]^ This makes the positive charge density of Cs^+^ more dispersed, weakening the coulombic force with OH^−^. Thus, regulating concentration, especially at CFC, reduces Cs^+^─OH^−^ association and increases electrical conductivity.

**Figure 2:**
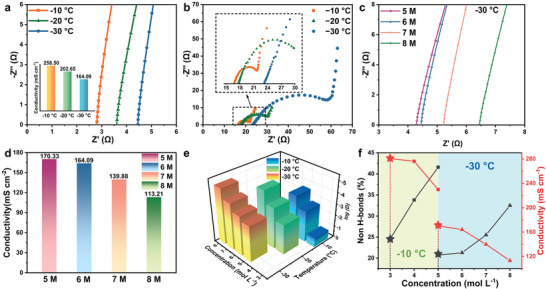
a) EIS spectra of the 6 m CsOH electrolyte. b) EIS spectra of AABs in 6 m CsOH electrolyte at −10 to −30 °C. c) EIS spectra of blocking electrodes of CsOH electrolytes ranging from 5 to 8 m at −30 °C. d) Ion conductivity of different electrolytes at −30 °C. e) The calculated diffusion constants of different concentration CsOH electrolytes from −10 to −30 °C. f) A summary graph depicting the variation of non H‐bonds and conductivity with concentration at low temperatures.

The optimized solvent structure would improve the batteries' performance with CFC. Thus, the temperature‐dependent ionic conductivities of 6 M CsOH electrolyte within the temperature range of −10 °C to −30 °C were studied, and the results are shown in **Figure** **2**a. Electrolyte conductivity is 258.50, 202.65, and 164.09 mS cm^−1^ at −10, −20, and −30 °C, respectively. There is an increasing trend in battery solution resistance and charge transfer resistance as the concentration is reduced (Figure 2b). This is due to the decrease in conductivity, which eventually leads to an increase in ohmic polarization.^[^ ^34^ ^]^ Therefore, enhancing electrolyte conductivity is expected to improve AABs performance. The ionic conductivity at the CFCs exhibits a maximum ionic conductivity. As shown in Figure 2c,d, and Figure S6 a,b (Supporting Information). At −30 °C, the electrolyte conductivity of 5 M CsOH surpasses 6 M (164.09 mS cm^−1^), 7 M (139.88 mS cm^−1^), and 8 M (113.21 mS cm^−1^). At −20 °C, 4 M (217.32 mS cm^−1^) CsOH exhibits maximal ionic conductivity. The order of ionic conductivity at −10 °C is 3 M > 5 M > 7 M. In addition, the diffusion constants of OH^−^ in these electrolytes can be qualitatively calculated by the mean‐squared displacement as a function of time (Figure S7a–c, Supporting Information)^[^ ^35^ ^]^:

(1) $$D=\lim_{t\to \infty }\frac{1}{6Nt}\left(\sum_{i=1}^{N}|r_{i}\left(t\right)-r_{i}\left(0\right){|}^{2}\right)$$

As shown in Figure 2e, the maximum diffusion constants at −30, −20, and −10 °C correspond to 5, 4, and 3 m electrolytes, which is in accordance with the variation trend of ion conductivity at low temperatures. The variation of non‐H‐bonds and conductivity with concentration at low temperatures was summarized. At different temperatures (Figure 2f), as the concentration of CsOH drops, the non‐H‐bonds decreases and the conductivity rises, affected by the OH^−^ de‐solvation and migration. Fast ion migration rate and water disorder are necessary for low‐temperature discharge of AABs.^[^ ^36^ ^]^ At −30 and −10 °C, the 5 and 3 m can reach extreme values respectively, corresponding to the CFCs. This predicts the best discharge capacity at CFCs.

**Figure 3:**
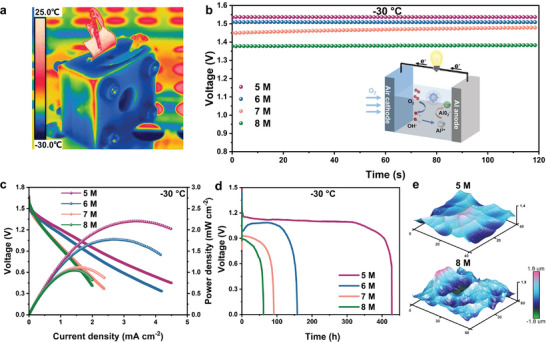
a) Infrared thermal imaging of AABs at −30 °C. b,c) The open circuit voltage and polarization curves of AABs with CsOH electrolytes from 5 to 8 m at −30 °C. The inset is the reaction mechanism diagram of AABs. d) The discharge curve of AABs working with CsOH electrolytes from 5 to 8 m at −30 °C. e) AFM images of Al anodes in 5 and 8 m CsOH electrolytes after discharge.

To confirm the above variation characteristics of CsOH as the cryogenic electrolyte, the performances of AABSs assembled by CsOH at CFCs were studied. The real‐time infrared thermal imaging under −30 °C is shown in **Figure** **3**a. First, the open‐circuit voltage and polarization were tested to evaluate battery performance at various temperatures and electrolyte concentrations. With the decrease in concentration, the open circuit voltage and power density rise gradually (Figures S8a,b and S9a,b, Supporting Information). Specifically, at the CFC of −30 °C (5 m), the open circuit voltage of the AABs is stable at ≈1.54 V (Figure 3b), which is superior to the other concentration. The maximum power density at −30 °C is 2.23 mW cm^−2^ (Figure 3c). At −30 °C, the discharge power density of AABs electrolyte in 5 m is twice that of 8 m. This implies that the AABs performance with CFC electrolyte is greatly improved compared with the concentrated electrolyte. Specifically, the battery with a high concentration of 8 m shows a continuous voltage decay and only operates for 61.4 h at 1 mA cm^−2^ at −30 °C (Figure 3d). For comparison, batteries with a CFC of 5 m can prolong the discharge time to 427.9 h. The AABs with CsOH of CFCs 3 m (−10 °C) and 4 m (−20 °C) can work continuously for 103.4 h and 68.4 h, respectively (Figure S10a,b, Supporting Information). Collectively, batteries under sub‐zero temperatures have the same trend. That is, the electrolytes under CFCs show the optimal discharge capability, including the power density and the stability duration. Additionally, the universality of the CFC rule was validated based on the different colligative properties of aqueous alkali. KOH and NaOH were selected and compared as alkaline electrolytes. As displayed in Figure S11a,b (Supporting Information), the CFC of KOH electrolyte for AABs analogous to CsOH is 4 m at −20 °C, with a stable discharge time of 17.4 h, higher than 8 m (14.5 h). The NaOH electrolyte at −20 °C only discharges for 5 min at 4 m, whereas the voltage at 8 m decays rapidly to 0 and cannot discharge normally. Therefore, CFC is suitable for AABs systems based on other alkaline electrolytes at sub‐zero temperatures.

**Figure 4:**
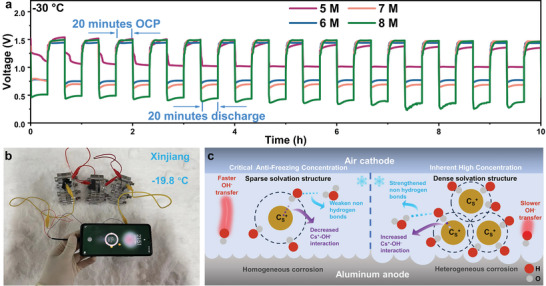
a) Voltage versus time for on/off cycling of batteries with CsOH electrolytes from 5 to 8 m at −30 °C. b) Charge a mobile phone on the snow in Xinjiang. c) The diagram of de‐solvation and corrosion mechanisms at critical anti‐freezing and inherent high concentration.

Regarding battery stability, the interfacial corrosion behavior of metal electrodes is also a decisive impact factor. With increasing concentration, the de‐solvation of OH^−^ becomes more difficult, leading to slow diffusion. Insufficient diffusion will cause concentration polarization and heterogeneous corrosion due to the accumulation of Al(OH)_3_ precipitates on the surface of the aluminum anode.^[^ ^37^ ^]^ The interface etching of aluminum was evaluated by Atomic Force Microscopy. The Al anodes assembled by 5 and 8 m CsOH were compared after discharge at −30 °C. Their relative roughness is 381 and 431 nm, respectively (Figure 3e). The lower surface roughness corresponds to homogeneous corrosion, resulting in a high utilization rate of aluminum plate.^[^ ^38^, ^39^, ^40^ ^]^ Moreover, the intermittent discharge experiments (20 min per cycle) were carried out at −30 °C. After 10 h of intermittent discharge, the battery with a low concentration of 5 m is stable, and the overall voltage is the highest (**Figure** **4**a), indicating the potential practical application of the CFC battery.^[^ ^41^ ^]^ The actual operation status of AABs in Xinjiang's winter night was also tested to evaluate the practical application, 5 m CsOH‐based AABs (three cells in series) can recharge a mobile phone at −19.8 °C (Figure 4b).

Furthermore, the evolution of solvated structures and Al corrosion are illustrated with CFC and higher concentrations (Figure 4c). In the entire system, a solvation structure with Cs^+^ as the core will be formed. The OH^−^ in the first shell is bonded to the Cs^+^ through Coulomb force and simultaneously forms a non‐H‐bond with the water in the second shell. When the electrolyte is concentrated, the metal interaction easily forms a large solvating group, a large amount of OH^−^ adsorbed in the second shell through hydrogen bonding, and finally hinders the ion migration. The free solvating group near the Al anode will cause uneven discharge. Conversely, Cs^+^ has a sparse solvated structure at the CFC attributed to decreased Cs^+^─OH^−^ and weak non‐H‐bonds. The OH^−^ in the first shell layer of Cs^+^ can escape easily and transfer faster, which can be sufficiently diffused to generate homogeneous corrosion. Unlike the inherent high concentration, the critical anti‐freezing concentration with the highest conductivity and without freezing is the most appropriate choice.

## 3 Conclusion

In summary, CsOH‐based electrolytes were employed to elucidate the kinetic process at the relatively lowest critical anti‐freezing concentrations (CFCs), which differs from the generally accepted high‐concentration electrolyte rule in aqueous electrolytes. The AABs with a CFC of 5 m CsOH deliver a longer discharge time of 427.9 h, a maximum conductivity of 170.33 mS cm^−1^ and homogeneous Al anode corrosion at −30 °C. The CsOH‐based electrolyte demonstrates a sparser solvation structure with CFCs, promoting the de‐solvation process of OH^−^ and producing more free OH^−^. The impact of the non‐H‐bonds in the solvation shell on the Cs^+^─OH^−^ association is reduced as the concentration decreases. This work not only demonstrates a strategy to operate the ABBs at sub‐temperature but also provides a new idea for the design of low‐temperature alkaline aqueous electrolytes.

## Conflict of Interest

The authors declare no conflict of interest.

## Supporting information

Supporting Information
