# Supplementary material for: How Thick Aqueous Alkali Should be Better for Aluminum‐Air Batteries at Sub‐Zero Temperatures: A Critical Anti‐Freezing Concentration
Source: Adv Sci (Weinh). 2024 May 30;11(29):2402005. doi: 10.1002/advs.202402005 (PMC11304294; doi:10.1002/advs.202402005)
Supplement: Supplementary file 1 — Supporting Information [file ADVS-11-2402005-s001.docx]

Supporting Information

How Thick Aqueous Alkali Should Be Better for Aluminum-air Batteries at Sub-Zero Temperatures: A Critical Anti-Freezing Concentration

*Hongyu Cui, Ming Gao, Guoqin Cao, Fanfan Liu, Junhua Hu^*^, Jinjin Ban^*^*

**1. Experimental Section**

**Electrode preparation**: The aluminum foil was purchased from a commercially available product with a purity of 99.99%. The aluminum alloy was mechanically polished firstly, then washed with deionized water and absolute ethanol in an ultrasonic cleaner for 5 min, respectively, finally dried in a vacuum at 70 °C for 8 h. The aluminum foil was subjected to mechanical polishing using sandpaper. Subsequently, it underwent sequential washing with deionized water and absolute ethanol in an ultrasonic cleaner for a duration of 10 minutes each. The treated aluminum foil was then dried under vacuum conditions at 55 °C for 5 h to obtain the anode material. The air cathode utilized in this study is a commercially available product, consisting of Pt/C catalyst and the conductive carbon cloth.

**Aluminum air batteries assembly**: The AABs were assembled following a stack-type cell configuration, and the cell volume was 3.768 cm^-3^. The air electrode was composed of carbon cloth (70×30 mm^-2^) with Pt/C catalyst. The loading area is 1 cm^-2^. The air electrode was modified by homogeneous catalyst ink. The mixed ink consists of 5 mg of commercial Pt/C catalysts, 570 μL mixed solution of ethyl alcohol, DI water and Nafion solution (5 wt %) with a ratio of 50:50:14. The anode is the polished aluminum foil electrode with a surface area of 70×30 mm^-2^ and a thickness of 0.3 mm. The AAB was assembled using an aqueous electrolyte consisting of 3-9 M CsOH and 5 M KOH.

**Characterization**: The structure properties of electrolytes were analyzed by using Raman (Horiba Scientific), NMR(Bruker Avance NEO 400MHz), and FTIR (Thermo Scientific Nicolet iS20). The freezing points were measured by Differential scanning calorimetry (DSC, NETZSCH DSC 214) from -60 to 20 °C at a scan rate at 5 °C min^-1^ in N_2_. Atomic Force Microscope (AFM 5100 N) was used to compare the surface morphologies of different electrolytic liquid systems. Surface analysis plays an important role in identifying the surface morphologies of aluminum anode in different electrolytes and the surface morphologies of different metals in the same electrolyte. Additionally, it facilitates exploring corrosion mechanisms for low-temperature performance analysis.

**Electrochemical measurements**: Electrochemical measurement was carried out in a conventional three-electrode cell by using a CHI 760 electrochemical workstation, which used an aluminum foil (10 mm × 10 mm × 3 mm) as the working electrode (WE), a Hg/HgO electrode as the reference electrode (RE) and platinum as the counter electrode (CE). The potentiodynamic polarization curves were varied from -1.8 to -1.2 V. The electrochemical impedance spectroscopy (EIS) experiments were performed at the OCP in the frequency from 100 kHz to 0.01 Hz with 10 mV amplitude. The electrochemical windows of the different electrolytes were obtained by linear sweep voltammetry (LSV) at 1 mV s^-1^ in the three electrode system. The ionic conductivity of the electrolyte was measured via the blocking electrode method using two stainless steel foils as the electrodes, the diagram is shown in **Figure S12**. Under temperature test chamber, EIS test was performed at 0.0 V over a frequency range from 10^-1^ to 10^5^ Hz with a voltage amplitude of 5.0 mV.

**Molecular dynamics simulations**: All molecular dynamic simulations were based on the LAMMPS package.^[1-2]^ The reactive force field potential developed by George M. Psofogiannakis et al.^[3]^ was used to describe the interaction between H, O, Cs, and K atoms, the conductivity of CsOH electrolytes at different concentrations and temperatures was theoretically explained from the perspectives of ion diffusion, bond length distribution and hydrogen bonding. The bond length truncation of hydrogen bonds is R_cut_ = 2.7 Å, with an angle truncation of 30°. The electrolyte systems were run at a constant temperature of one million steps under the NVT (NVE+berendsen temperature control^[4]^) ensemble, and the time step is 0.1 fs.

The pair distribution function represents the average probability of finding $\beta$ particle in the unit volume of the spherical shell at the distance of $r$ from the$\alpha$ central particle. It is the Fourier transform of the structure factors obtained from X-ray diffraction, which is an important structural parameter for comparing theoretical models with experimental results. It is a statistical parameter that reflects the characteristics of the architecture, which is defined as:

$g_{\alpha\beta}(r)=\frac{N}{4\pi\rho N_{\alpha}N_{\beta}}\sum_{i}^{N_{\alpha}} \sum_{j=1,j\neq i}^{N_{\beta}} \delta(r-\left| \overset{\to}{r_{ij}} \right|)$ (1)

In formula (1), $N$ is the total number of particles in the system, and $\rho$ is the average atomic number density of the system. $N_{\alpha}$ and $N_{\beta}$ are the number of $\alpha$ and $\beta$ atoms in the system, respectively. $\left| \overset{\to}{r_{ij}} \right|$ is the distance between the number i atom ($\alpha$) and number $j$ atom ($\beta$). If the atomic species of the system are not distinguished or if it is a monatomic system, the expression degenerates to:

$g(r)=\frac{\left\langle\rho(r) \right\rangle}{\rho}=\frac{1}{4\pi\rho N}\sum_{i}^{N} \sum_{j=1,j\neq i}^{N} \delta(r-\left| \overset{\to}{r_{ij}} \right|)$ (2)

**Aluminum air battery tests**: The linear sweep voltammetry curves were measured with a sweep rate of 0.01 mV s^-1^ and a voltage range of 0 to 1.5 V. The power density (P) of the aluminum air battery was determined using the equation: P = I·V, where I represents the discharge current density and V corresponds to the voltage. The discharge performance of the aluminum-air battery was evaluated at various current densities ranging from 1 to 5 mA cm^-2^ within a temperature-controlled chamber set to -10 °C to -30 °C.

**2. Supplementary Results and discussion**

In theory, icing requires a certain degree of supercooling to initiate the critical ice core,^[5]^ which is closely related to the content of impurities, bubbles or solid particles generated by electrochemical reactions in the solution. Therefore, the production of reaction products and hydrogen bubbles during the discharge of AABs and the decrease in alkali concentration may affect the freezing point. In addition, external fields such as mechanical force fields, electric fields, and magnetic fields during the discharge process can also influence the nucleation and growth of ice. This discovery is visually presented in Figures S1b and c, the freezing point of the electrolyte will increase after discharge, this phenomenon may be related to the nucleation theory of Figure S1d, after AABs discharge, the electrolyte tends to undergo heterogeneous nucleation, with more nucleation sites acting as the starting points for ice crystal formation. This reduction in the energy barrier of nucleation promotes the ordered arrangement of surrounding water molecules, thereby facilitating the aggregation of water molecules to form larger ice crystals.^[6]^

In order to ensure that the exploration process is not disturbed by freezing factors, subsequent electrochemical experiments and simulation calculations were conducted through different concentrations electrolytes of 3 M, 4 M, and 5 M CsOH, which will be the lowest concentrations at -10 °C and -20 °C and -30 °C, respectively.

**3. Supporting Figures**





**Figure S1.** a) Definition of critical anti-freezingconcentration and variation with temperature and concentration. b-c) The optical photographs of different electrolytes concentrations at low temperatures after and before discharge. d) Schematic diagram of icing nucleation.


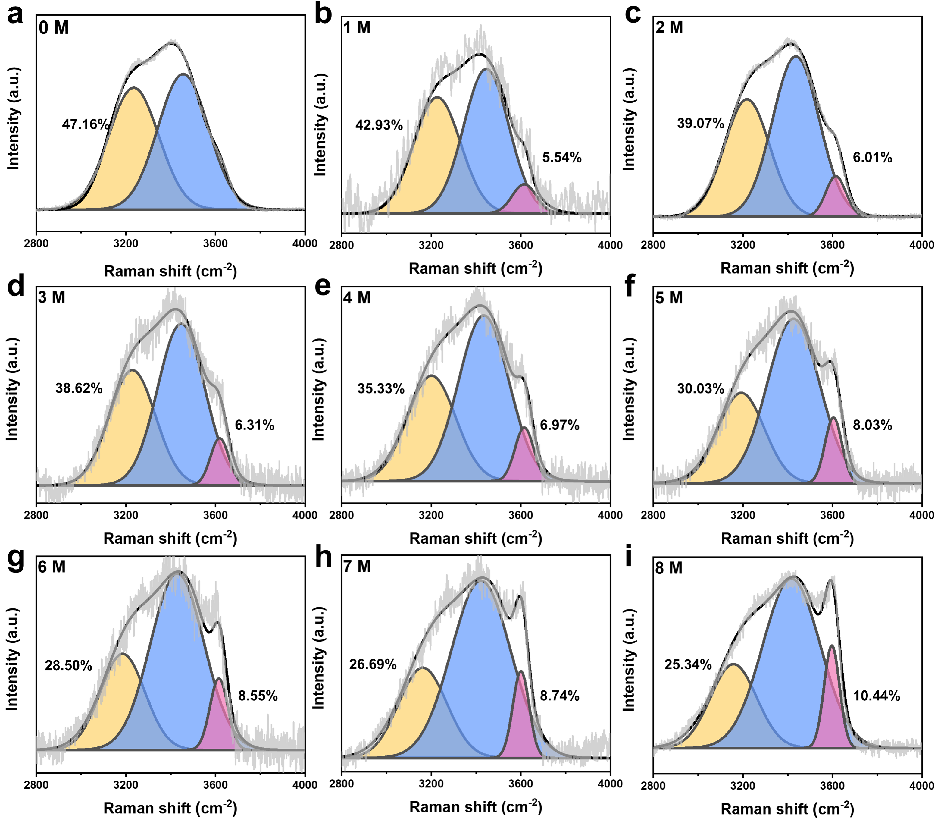


**Figure S2.** The fitted OH stretching vibration of electrolyte with CsOH from 0 to 8 M with strong, weak, and non H-bonds.


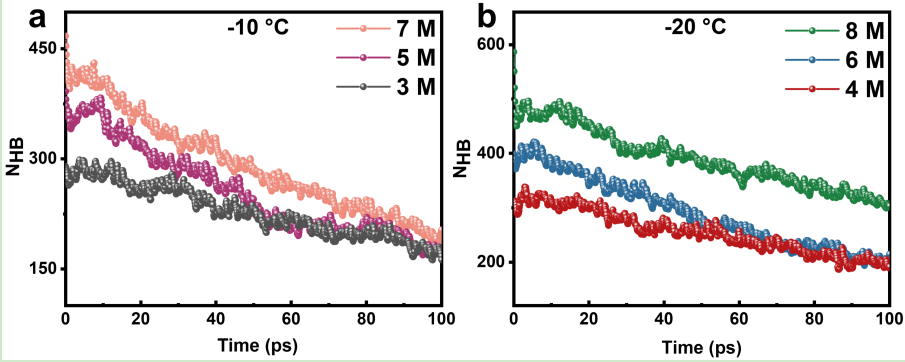


**Figure S3.** The average non H-bonds number of CsOH electrolytes with different concentrations at -10 °C a) and -20 °C b).


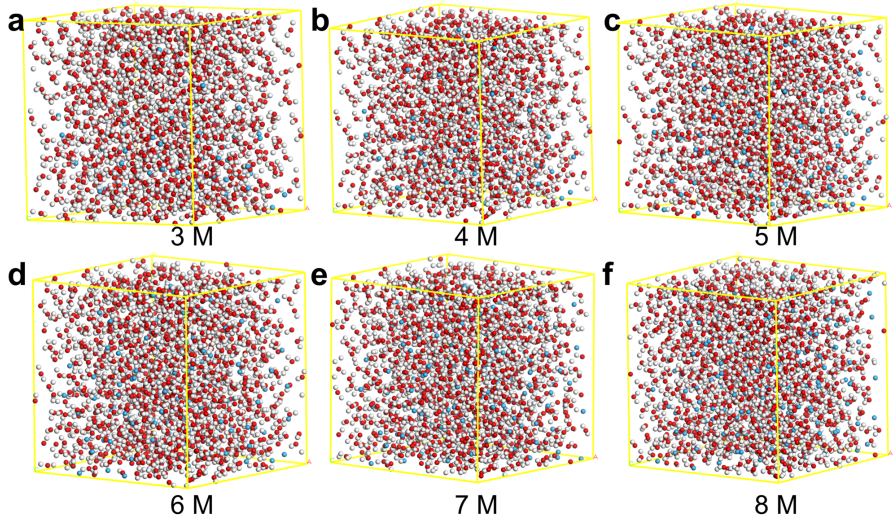


**Figure S4**. Snapshots of the MD simulation boxes containing CsOH electrolytes of various concentrations. Colors for different elements: H grey, O red, Cs blue.


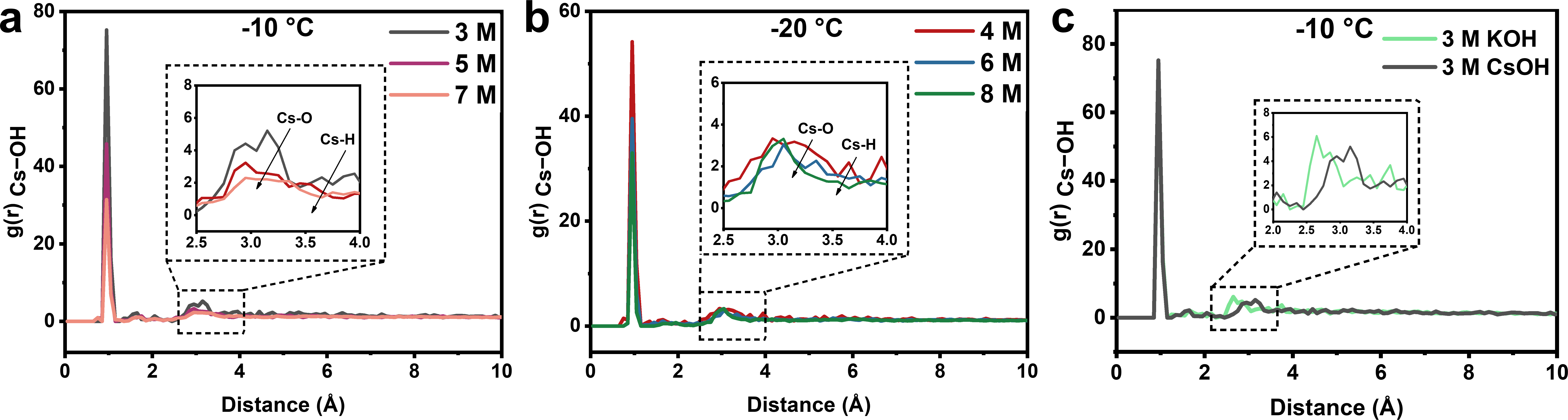


**Figure S5.** a) Cs-OH radical distribution function at -10 °C. b) Cs-OH radical distribution function at -20 °C. c) Cs-OH and K-OH radical distribution function comparion at -10 °C.


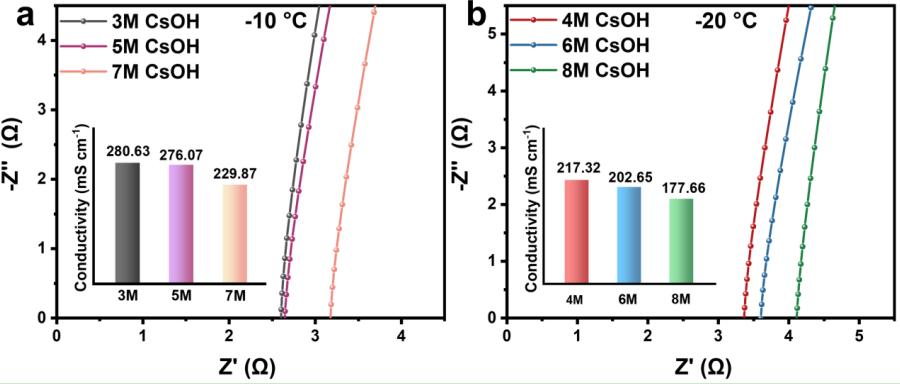


**Figure S6.** EIS spectra of different concentration CsOH electrolytes blocking electrodes at -10 °C a) and -20 °C b).


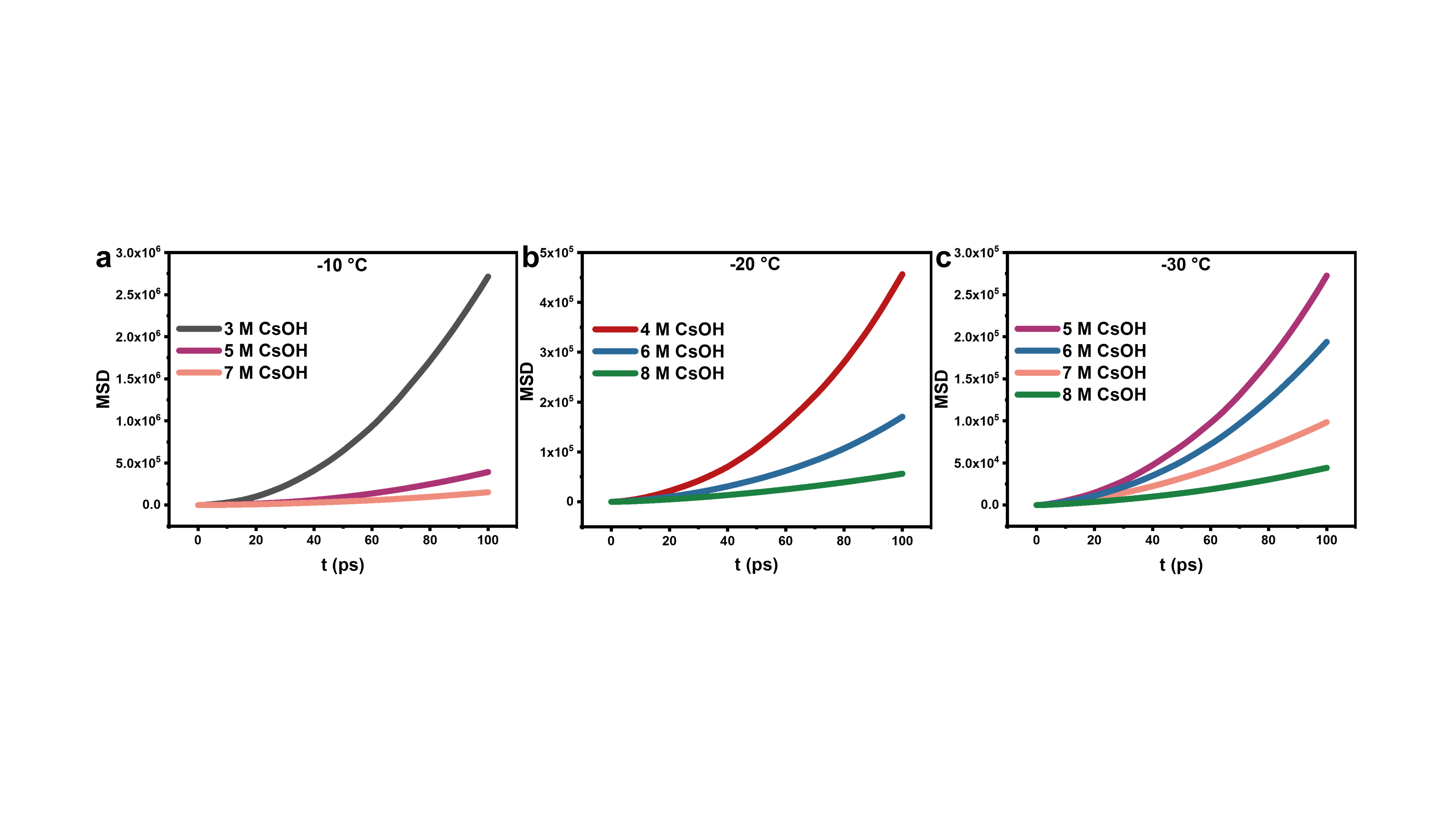


**Figure S7** a) The diffusion rate of OH^-^ with different concentration of CsOH at -10 °C. b) The diffusion rate of OH- with different concentration of CsOH at -20 °C. c) The diffusion rate of OH- with different concentration of CsOH at -30 °C.


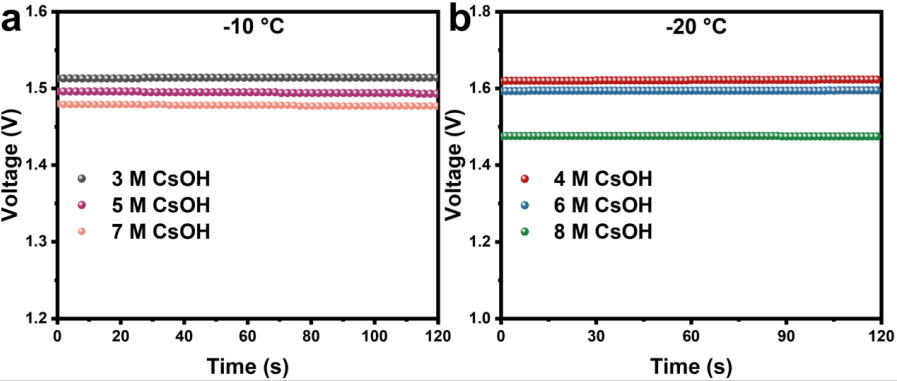


**Figure S8** The open circuit voltage of AABs working with different CsOH electrolytes at -10 °C a) and -20 °C b).


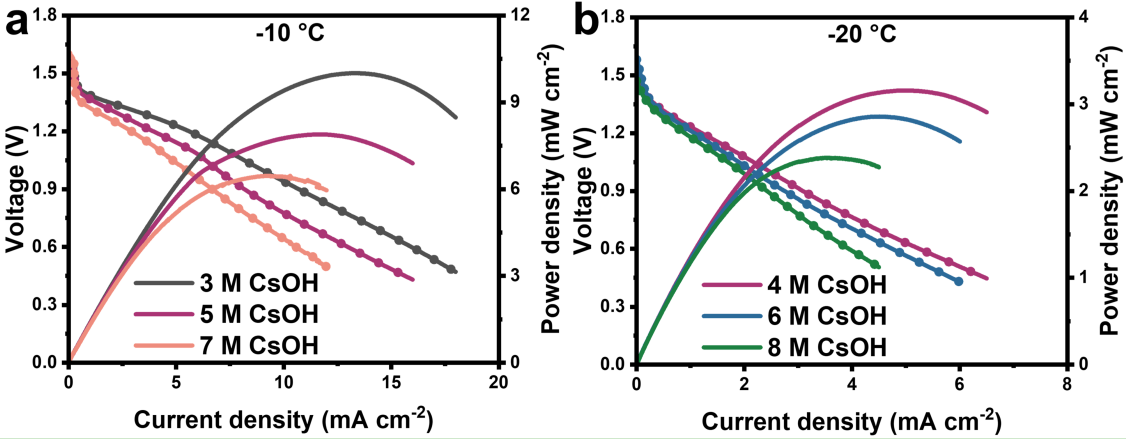


8

**Figure S9** The polarization curves of AABs working with different CsOH electrolytes at -10 °C a) and -20 °C b).


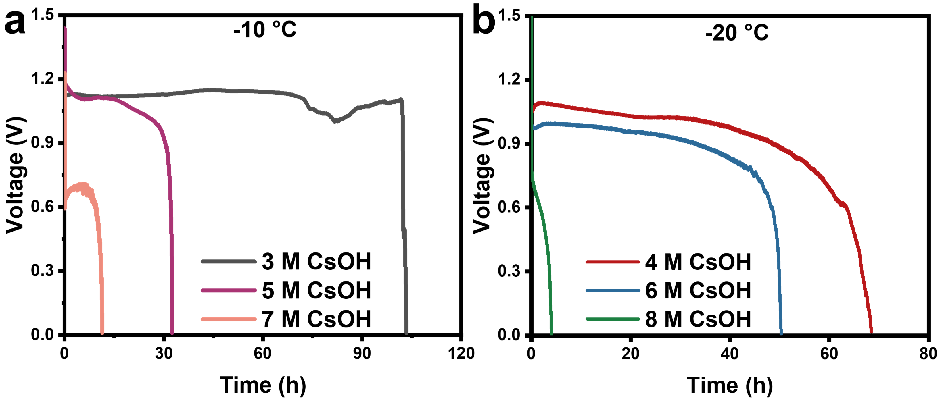


**Figure S10** The discharge curves of AABs working with different CsOH electrolytes at -10 °C a) and -20 °C b).


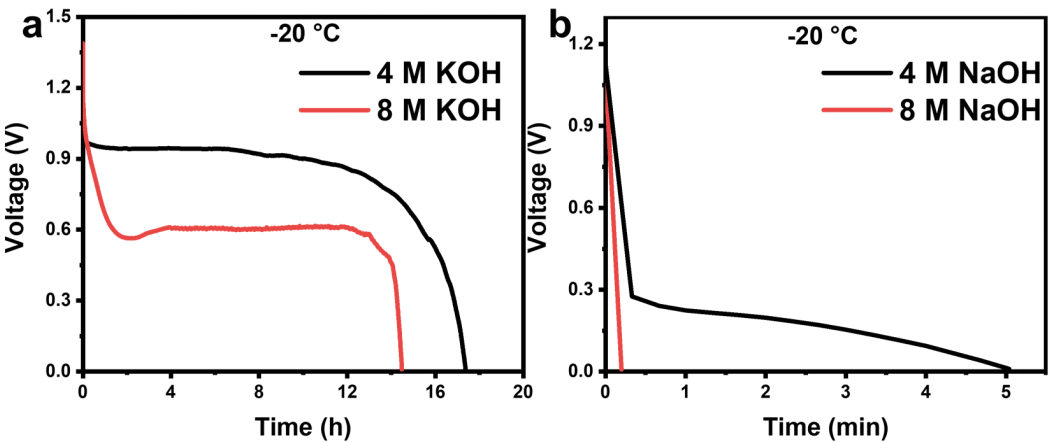


**Figure S11** a) The discharge curves of batteries working with 4 M and 8 M KOH electrolytes at -20 °C. b) The discharge curves of batteries working with 4 M and 8 M NaOH electrolytes at -20 °C.


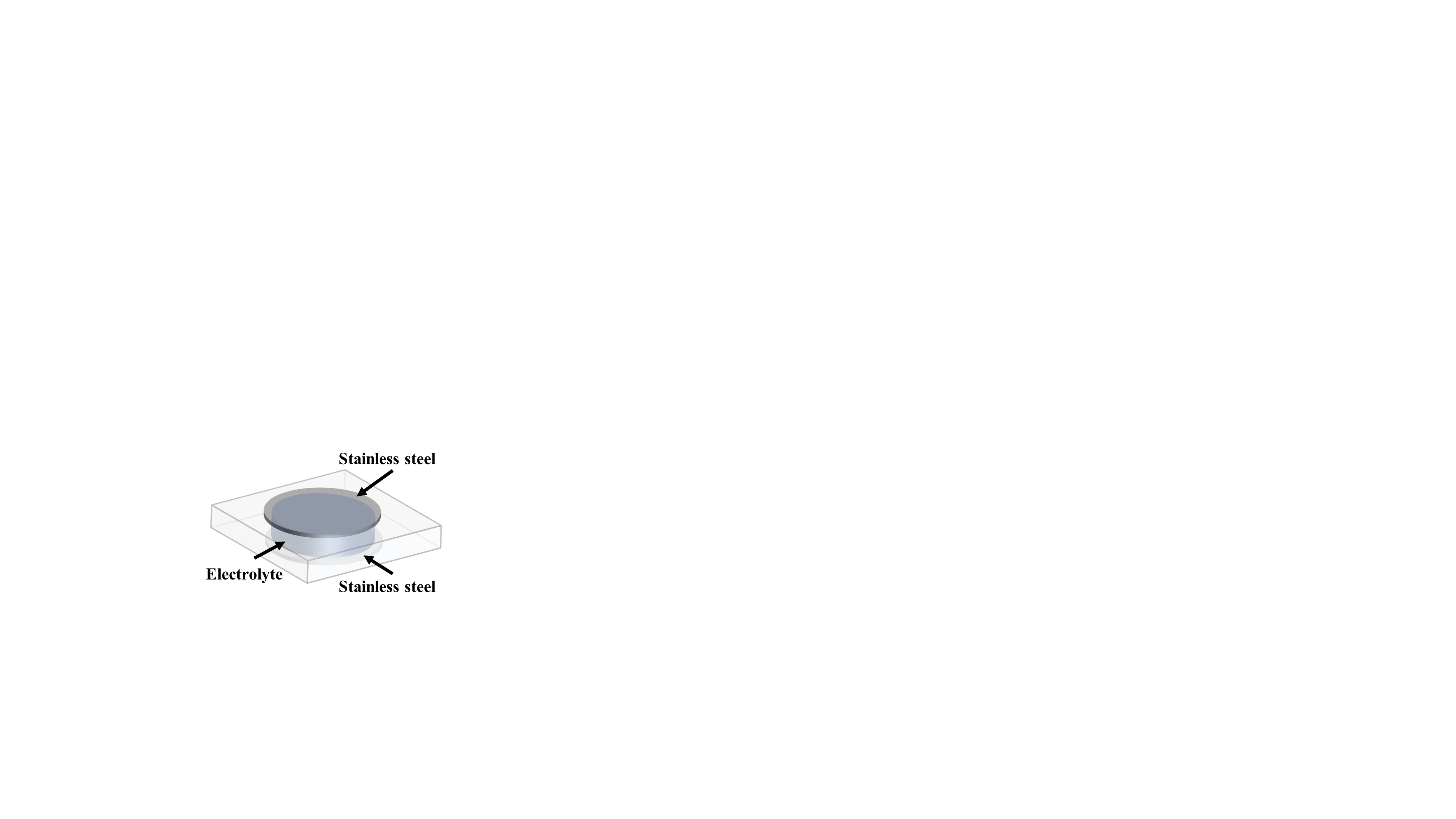


**Figure S12** Conductivity test blocking electrode mold schematic diagram.

**Table S1**. The proportion of CsOH at each concentration was fitted to strong, weak, and nonH-bonds at around 3000~3700 cm^-1^

| C_CsOH_ (mol L^-1^) | Strong H-bonds (%) | Weak H-bonds (%) | Non H-bonds (%) |
| --- | --- | --- | --- |
| 0 M | 47.16 | 52.84 | 一 |
| 1 M | 42.93 | 51.53 | 5.54 |
| 2 M | 39.07 | 54.92 | 6.01 |
| 3 M | 38.62 | 55.07 | 6.31 |
| 4 M | 35.33 | 57.70 | 6.97 |
| 5 M | 30.03 | 61.94 | 8.03 |
| 6 M | 28.50 | 62.95 | 8.55 |
| 7 M | 26.69 | 64.57 | 8.74 |
| 8 M | 25.34 | 64.22 | 10.44 |

[1] S. Plimpton, *J. Comput. Phys.* **1995**, *117*, 1-19.

[2] L. Verlet, *Health Phys.* **1967**, *22*, 79-85.

[3] G. M. Psofogiannakis, J. F. Mccleerey, E. Jaramillo, A. C. T. V. Duin, *J. Phys. Chem. C.* **2015**, *119*, 6678-6686.

[4] H. J. C. P. Berendsen, J. P. M. V. Postma, W. F. V. Gunsteren, A. D. Dinola, J. R. Haak, *J. Chem. Phys.* **1984**, *81*, 3684.

[5] K.-J. Wu, E. C. M. Tse, C. Shang, Z. Guo, *Prog. Mater Sci.* **2022**, *123*, 100821.

[6] G. Bai, D. Gao, Z. Liu, X. Zhou, J. Wang, *Nature* **2019**, *576*, 437-441.
